# The Spectrum of Hemolytic Disease of the Newborn: Evaluating the Etiology of Unconjugated Hyperbilirubinemia Among Neonates Pertinent to Immunohematological Workup

**DOI:** 10.7759/cureus.16940

**Published:** 2021-08-06

**Authors:** Suman S Routray, Rachita Behera, Bhabagrahi Mallick, Devi Acharya, Jagdish P Sahoo, Girija N Kanungo, Bibudhendu Pati

**Affiliations:** 1 Transfusion Medicine, All India Institute of Medical Sciences, Bhubaneswar, Bhubaneswar, IND; 2 Department of Transfusion Medicine, Institute of Medical Sciences and SUM Hospital, Bhubaneswar, IND; 3 Department of Pediatrics, Institute of Medical Sciences and SUM Hospital, Bhubaneswar, IND; 4 Department of Transfusion Medicine, AMRI Hospitals, Bhubaneswar, IND; 5 Department of Neonatology, Institute of Medical Sciences and SUM Hospital, Bhubaneswar, IND

**Keywords:** neonatal jaundice, direct antiglobulin test, elution, phototherapy, exchange transfusion

## Abstract

Background and objective

The exact burden of hemolytic disease of the newborn (HDN) attributed to neonatal unconjugated hyperbilirubinemia (NUH) in developing nations is still unclear. Still, anti-D is reported to be the most common cause of HDN in India. ABO incompatibility has emerged as a leading cause of exchange transfusion (ET) in many countries. But many centers in our country rely on direct antiglobulin test (DAT) as a screening tool to evaluate immunological causes, whereas advanced immunohematological workup like antibody screening, identification, and elution tests are also required. Early identification of implicated antibodies resulting in HDN can aid in the proper selection of blood units when ET is indicated, and hence also in managing the subsequent pregnancy. This study focused on determining the causes of neonatal hyperbilirubinemia (NH), especially with respect to immunohematological evaluation. This cross-sectional study was conducted on 240 neonates requiring neonatal intensive care unit (NICU) support for NUH at a tertiary care hospital.

Materials and methods

Demographic data, along with detailed history pertaining to the cause of hyperbilirubinemia, was collected. Clinical and laboratory evaluation and complete immunohematological work including DAT, heat elution, antibody screening, antibody identification, and Rh Kell phenotyping were performed from neonate blood samples. Data were analyzed using SPSS Statistics version 19 (IBM Corp., Armonk, NY).

Results

Pathological jaundice was more common (62.1%) than physiological jaundice (37.9%). The various pathological causes identified were HDN (42.6%), sepsis (12%), cephalohematoma (5.4%), and idiopathic (1.7%). Among HDN cases, ABO incompatibility (39.2%) was the most prevalent cause, followed by Rh HDN and G6PD deficiency (1.7% each). DAT was positive in only 14 cases out of 94 ABO-incompatible cases. Elution revealed antibodies in four DAT-negative neonates with ABO incompatibility and more specificity to the OA setting. DAT was positive with 100% sensitivity in Rh HDN cases (n=4). Elution demonstrated the presence of anti-D (n=2), anti-D + anti-C (n=1) and anti-E (n=1), confirming Rh HDN. DAT strength was found to be significantly associated with hemoglobin (Hb) level (p=0.048). The majority of cases were treated with phototherapy only (94.1%), and 10 cases received both ET and phototherapy. Four neonates' condition improved without any intervention.

Conclusion

This study highlighted the shift in the trend from Rh HDN to ABO incompatibility as the cause of hemolytic jaundice in NICU neonates. Elution tests can aid in the diagnosis of DAT-negative ABO-incompatible hemolytic anemia. Early diagnosis, along with timely intervention and appropriate measures, can prevent neonatal morbidity and mortality. Negative DAT does not rule out HDN. Sensitive techniques like elution must be used in the presence of clinical suspicion.

## Introduction

Neonatal hyperbilirubinemia (NH) is a commonly recognized global health problem often warranting readmission to hospitals, and it is associated with a high morbidity and mortality rate in low-income and middle-income countries [1,2]. It refers to elevated serum bilirubin concentration in the neonates resulting in yellowish discoloration of skin and sclera of eyes. The serum bilirubin level required to cause jaundice among neonates varies with skin tone and body region. Jaundice typically progresses in a cephalocaudal direction and is usually detected on the sclera at total serum bilirubin (TSB) level of 2-3 mg/dL (34-51 μmol/L). About 25-50% of all term neonates and a higher percentage of preterm babies develop clinical jaundice during the neonatal period, requiring complete medical evaluation and often admission to neonatal intensive care unit (NICU) [3]. Though neonatal jaundice is often a benign and transient physiologic consequence of the newborn's immature liver, various other medical conditions can cause severe neonatal jaundice. Permanent brain damage may occur due to excessively elevated levels of unconjugated bilirubin (a condition called kernicterus). Early diagnosis and timely interventions such as phototherapy and exchange transfusion (ET) will reduce the risk of neonates developing kernicterus. Immunological and non-immunological causes attributed to NH are well described in the literature, but there is scarce data on immunological findings in neonates. Although many Indian studies have revealed the different antibodies implicated in causing hemolytic disease of the newborn (HDN) in the antenatal settings, we could hardly find any literature describing immunohematological findings in neonates. Moreover, antenatal screening is focused chiefly on Rh-negative mothers, and screening for ABO HDN is not routinely done in our country. In light of this, the present study was conducted to analyze various causes of unconjugated hyperbilirubinemia in neonates admitted in the NICU of a tertiary care hospital. The primary focus of the study was to evaluate the immunological causes and ascertain the antibodies responsible for HDN.

## Materials and methods

This study was conducted in the NICU of a tertiary care hospital in Odisha for 18 months (December 2015 to May 2017). The Institutional Ethics Committee approved the study. This cross-sectional study included a total number of 240 neonates with unconjugated hyperbilirubinemia admitted to NICU. Neonates with a congenital malformation, those who left against medical advice, those whose parents refused to enroll in the research, and those whose mothers had a maternal history of autoimmune hemolytic anemia were excluded from the study. After explaining the study's purpose, written informed consent was obtained from all the participating parents. At any stage during the study period, parents were allowed to discontinue on their own accord.

Complete demographic data of the neonate, detailed history related to the causes of neonatal jaundice, such as gestational age, birth weight as per the World Health Organization (WHO) classification, history of exclusive breastfeeding, cephalohematoma, complete maternal obstetric history, any previous premature rupture of membrane, neonatal jaundice in the last child, oxytocin during the present birth, details of receiving Rh Ig immunoprophylaxis during the last or current pregnancy, blood transfusion history, and any associated comorbidities were recorded.

This was followed by a clinical evaluation with a particular focus on assessing hyperbilirubinemia severity. Complete blood count (CBC) using SYSMEX 100 automated analyzer (Sysmex Corporation, Kobe, Japan), peripheral blood picture, reticulocyte count, liver function test (LFT) using Roche Cobas 6000 analyzer (Roche Holding AG, Basel, Switzerland), forward blood grouping (with monoclonal antisera, Tulip Diagnostics, Mumbai, India), immunohematology (IH) workup, glucose-6-phosphate dehydrogenase (G6PD) level estimation, thyroid profile [free triiodothyronine (FT3), free thyroxine (FT4), thyroid-stimulating hormone (TSH)], c-reactive protein (CRP), blood culture, erythrocyte sedimentation rate (ESR), and serum procalcitonin were performed to ascertain the cause contributing to it.

Immunohematology workup

Both forward and reverse blood grouping (using pooled A, B, and O cells) of the mother (if required, the father's too) was performed. Rh-negative status of the mother was confirmed using anti-D monoclonal antisera of two different lot numbers. Both polyspecific and monospecific direct antiglobulin test (DAT; using IH card, Tulip Diagnostics) was performed on neonates' blood samples. Heat elution was performed on neonates' RBCs irrespective of the DAT status. The eluate was tested against pooled A, B, and O cells and commercially available cell panels (Diacell I-II-III & ID-Diapanel, Biorad, Switzerland) to determine the specificity of the implicated antibody. Antibody screening and identification of the mother were performed using the same Diacell/Diapanel as and when feasible (many patients were referred with the mothers being treated in the primary center). Phenotyping of red cells (IH Rh-Kell card, Biorad) was performed on neonates and parents' blood samples to confirm the offending antibody (antibody to Rh C, c, E, e, and K) responsible for HDN. All the immunohematological workup was done according to the American Association of Blood Banking (AABB) technical manual.

The severity of the condition was analyzed based on TSB, and treatment was initiated as per the American Academy of Pediatrics (AAP) guidelines. Extreme hyperbilirubinemia was defined as a TSB level of ≥25 mg/dl.

Double volume exchange transfusion (DVET) was performed using irradiated, leukodepleted, saline-adenine-glucose-mannitol (SAGM)-free, antigen-negative blood units suspended in AB plasma and compatible with both mother and neonate. Generally, the RBC unit volume and AB plasma were mixed in a ratio of 7:3 to get a hematocrit of 55-65% in the reconstituted unit. Antigen negative refers to the absence of blood group antigen(s) in the packed RBC (pRBC) unit against the corresponding detected antibody or antibodies in the neonate [4].

Data analysis

Statistical analysis was performed with SPSS Statistics software version 19.0 (IBM Corp., Armonk, NY). Categorical data were presented as proportions. The Shapiro-Wilk test was used to assess the normality of the data. The measure of central tendency was taken as the median and interquartile range in the skewness of the data. Non-parametric tests like Spearman's rank correlation were used to assess the relationship between variables. A Chi-square test was used for unrelated categorical data. A p-value of ≤0.05 was taken as statistically significant with a confidence interval of 95%.

## Results

In this study, out of 240 neonates with NH, 140 (58.3%) were male and 100 (41.7%) were females; 173 (72.1%) were term, and 63 (26.3%) were preterm neonates. The gender distribution, among term, near-term, and preterm neonates, is depicted in Table 1.

**Table 1:** Demographic details of neonates with reference to gender

Gestational age	Male	Female	Total
	Number (%)	Number (%)	
Term (≥37 weeks)	96 (55.5%)	77 (44.5%)	173
Near-term (34-37 weeks)	3 (75.0%)	1 (25.0%)	4
Preterm (<34 weeks)	41 (65.1%)	22 (34.9%)	63
Total	140 (58.3%)	100 (41.7%)	240

**Figure 1:**
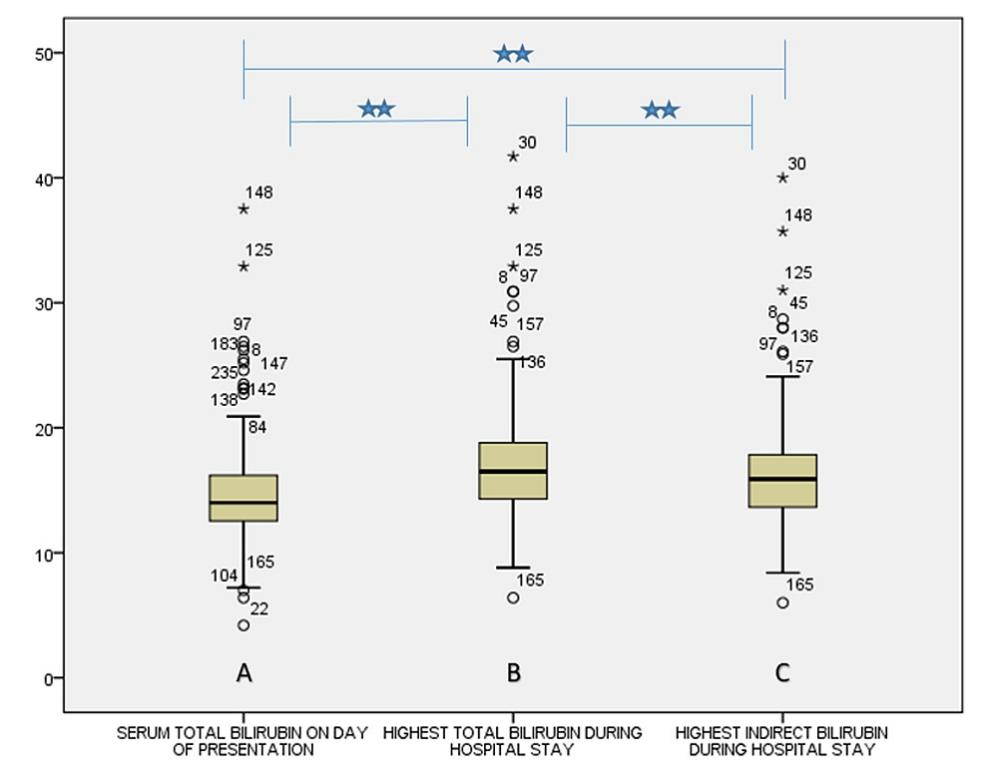
Box plots depicting (A) the serum total bilirubin levels on the day of presentation to the hospital, (B) highest serum total bilirubin level during the hospital stay, and (C) highest serum indirect bilirubin levels during the hospital stay Note: there was a significant correlation (two-tailed) at 0.01 level as assessed by Spearman’s correlation among the three panels

The majority of the neonates (n=204, 85%) were presented to NICU between two to five days of birth. The rest of the cases were presented as follows: four cases within 24 hours, 26 patients between 5-10 days, and six cases after 10 days. The highest level of TSB on the presenting day was 37.5 mg/dl [median: 14, interquartile range (IQR): 3.67], whereas during the hospital stay, it was 40 mg/dl (median: 16.5, IQR: 4.50) and that of peak indirect bilirubin was 39.50 mg/dl (median: 15.9, IQR: 4.25). There was a statistically significant correlation between TSB on the day of presentation and peak TSB (p=0.000) and peak indirect bilirubin (p=0.000) during the hospital stay (Figure 1).

Pathological jaundice was more common (n=149, 62.1%) than physiological jaundice (n=91, 37.9%). Among the pathological cases, incompatibility (39.2%) was the most prevalent cause, followed by sepsis (12%), and cephalohematoma (5.4%); Rh HDN, G6PD deficiency, and idiopathic causes contributed 1.7% of cases each in the present study. Only one case (0.4%) presented with both ABO (anti-B) and Rh HDN (anti-c).

We encountered 94 ABO-incompatible cases with clinical and laboratory evidence of hemolysis along with peripheral smear examination suggestive of ABO HDN, as depicted in Table 2.

**Table 2:** Distribution by day of presentation of cases of ABO hemolytic disease of the newborn

Diagnosis	Day of presentation			Total
	2-5	6-10	>10	
	N	N	N	N
OA setting	30	2	1	33
OB setting	49	2	1	52
AB setting	1	1	0	2
BA setting	6	1	0	7
Total	86	6	2	94

DAT was positive only in 14 cases (14.9%), predominantly in A group neonates born to O group mother (OA setting). Among DAT-positive cases, eight (57.1%) were weakly positive, and six (42.9%) were strongly positive. Eluate was positive for anti-A (n=3) or anti-B (n=1) in four affected neonates, with DAT being negative. A statistically significant correlation was observed between DAT and eluate. The maximum number of ABO HDN cases (n=87) presented between days two to five of birth. Out of 94 ABO HDN cases, 56 (58.9%) were born to primigravida mothers.

Rh alloimmunization was detected in five cases, and in all these cases, DAT was strongly positive. Anti-D (n=2), anti-D and anti-C (n=1), anti-E (n=1), and anti-c (n=1) were demonstrated in both neonate and maternal serum. Three cases manifested within 24 hours of birth. There was a statistically significant positive correlation between multiparity and Rh HDN (p=0.002). Anti-c and anti-E were found in Rh-positive mothers with bad obstetric history.

The hemolysis severity based on hemoglobin (Hb) level was compared with gestational age, gender, and DAT strength, as depicted in Table 3.

**Table 3:** Association of hemoglobin levels with gestational age, gender, and direct antiglobulin test strength a: term; b: near-term; c: preterm; DAT: direct antiglobulin test; M: male; F: female

		Hemoglobin level (gm/dl)					P-value
		<12.5	12.5-17			>17	
Gestational age	a	13		88	72		0.390
	b	1		3	0		
	c	5		31	27		
Gender	M	12		73	55		0.760
	F	7		49	44		
DAT strength	0	16		108	98		0.048
	1	0		1	0		
	2	1		5	1		
	3	2		7	0		
	4	0		1	0		

DAT strength was found to be significantly associated with Hb level (p=0.048). Hb level, bilirubin level, reticulocyte count, and DAT strength were correlated significantly in two-tailed Pearson correlations (p=0.000).

Most of the cases were treated with phototherapy only (226, 94.1%), and 10 patients received both ET and phototherapy.

## Discussion

This study revealed a changing trend in etiology contributing to neonatal jaundice in a developing nation where a significant number of cases were due to ABO incompatibility as observed in developed countries like the USA or Canada, using advanced investigations. The prevalence of pathological jaundice (62.1%) was found to be more than physiological jaundice (37.9%). This might be due to the inclusion of neonates admitted to NICU in the study. Studies from high-income countries have revealed that ABO incompatibility was the single most leading cause of severe NH [5,6]. Rh-negative pregnancy, septicemia, malaria, and G6PD deficiency contribute to significant causes in low-income and middle-income countries [7,8]. ABO incompatibility was the most important etiological factor attributed to hemolytic hyperbilirubinemia, presenting between two to five days of birth (91.6%) and more commonly with B group neonates (42.5%) born to O group mothers. Literature has revealed that neonates with the A blood group are being more widely affected [9]. The difference could be due to variation in blood group distribution among different regions and ethnicities. In ABO incompatibility cases, though 58.9% were firstborn, parity had no significant association, as per the study conducted by Dufour and Monoghan [10].

In our study, male babies (58.3%) were found to be more affected than female babies (41.7%). The male preponderance in the study may be due to existing social bias that encourages preference towards males and the fact that parents immediately seek medical attention for their male children. Our study population comprised 26.3% of preterm neonates. The immature bilirubin conjugating system, increased enterohepatic circulation, decreased caloric intake, and a higher hemolysis rate make preterm neonates more prone to develop NH [11].

The maximum number of cases (85%) with NH presented to our hospital between two to five days of birth. This is probably because of ABO incompatibility, which usually presents between two-five days of delivery, as suggested by various studies in the literature [11]. Only 26 (10.8%) cases presented between 6-10 days of birth. Hyperbilirubinemia was observed in four patients within 24 hours of delivery and in six instances after the 10th postnatal day. There are factors that could explain the delayed presentation, like outborn neonates, sepsis, and low maternal educational status [12]. The very early presentation (within 24 hours of birth) was due to Rh HDN and G6PD deficiency in our cases.

The mean serum bilirubin was 15.33 mg/dl on the presenting day. The highest serum bilirubin levels and highest serum indirect bilirubin levels during hospital stay were 18.16 mg/dl and 17.9 mg/dl, respectively, among the ABO HDN cases. We found a strong positive association between serum bilirubin on the presenting day and maximum bilirubin reached during the hospital stay (p=0.000). Similar findings were also observed by Cariani et al. [13]. The mean Hb level was 15.51 gm/dl among the ABO HDN neonates. There was a significant negative correlation between Hb level and serum bilirubin level on the presenting day (p=0.001), as supported by the studies of Cariani et al. and Olivares et al. [13,14].

DAT was positive in only 14.9% (14/94) of cases, predominantly in neonates with OA setting (8/14). Low expression of A and B antigens over neonatal red blood cells may be the reason for the low DAT positivity. Moreover, ABO antigens are widely distributed over tissues other than RBCs, resulting in decreased binding of these antibodies to RBCs and less severe hemolysis [15,16]. Studies from New Zealand, Greece, and India [17-19] have reported that the incidence of DAT positivity was slightly higher in neonates with OA setting when compared to OB setting. Hemolysis by anti-A is more common (one in 150 births) than anti-B, and this may explain the presence of more DAT-positive cases among A group neonates [9] compared to black African-origin neonates who exhibit more of the OB setting [20]. In an OB setting with a positive DAT, one case showed positive antibody screening (anti-c) in maternal and neonatal serum. DAT positivity was attributed to irregular antibodies and ABO incompatibility, as confirmed by the elution test. We encountered one case of DAT positivity in the BA setting. Elution was positive for the corresponding antibody in all DAT-positive samples. Elution demonstrated the presence of antibodies in four DAT-negative ABO HDN cases. This may be attributed to the fact that antibody concentration is increased by elution [21]. We observed a strong positive correlation of DAT with heat elution (p=0.000).

In this study, we encountered 21 cases of Rh D incompatibility, out of which four cases had laboratory features of hemolysis and was confirmed by the presence of anti-D (two cases), anti-D + anti-C (one case), anti-E (one case) in both maternal and neonatal serum by antibody screening. DAT was positive in all four cases. In a developing nation, anti-D is the most common alloantibody present in the Rh-negative alloimmunized mother. In the USA, anti-D + anti-C is the most common combination in women with multiple RBC antibodies, and they are more likely to develop significant HDN [22]. Rh HDN with negative DAT has also been reported in the literature [23]. We encountered one case of ABO incompatibility along with Rh HDN. All the cases of Rh HDN were observed in multigravida mothers (100%). A significant correlation was found between positive DAT and parity among Rh-negative mothers (p=0.009), which was, as per the findings of Jeremiah et al. [24], because of previous sensitization.

HDN (n=6) followed by sepsis (n=4) was a major cause requiring ET in our setting. ABO HDN in OB setting (n=2), Rh HDN (n=2) due to anti-D + anti-C and anti-E, ABO + Rh HDN (n=1), and G6PD deficiency (n=1) were attributed to ET in HDN. Studies from the Middle East and Southern Europe have reported that ABO and Rh HDN manifest as severe hyperbilirubinemia requiring ET [25,26]. In contrast, in a study from South Africa, iso-immune hemolysis is attributed to only a fraction (14%) of ET [27].

G6PD deficiency was attributed to only 1.7℅ cases and manifested only in males. These findings were similar to studies from other parts of India but at a much lower frequency than African American babies [28,29]. Interestingly, G6PD deficiency-induced hemolytic anemia has also been reported in heterozygote females despite the typical X-linked inheritance pattern [30]. In two NH cases where antibody screening in neonates was negative, the maternal serum demonstrated Anti-Lea. Failure of Anti-Lea antibody to cause HDN may be due to less developed antigens over neonatal red cells or antibodies' neutralization by fetal antigen in the placenta. No etiological factor for the NH could be established in four (1.7%) neonates, much lower than other studies using advanced IH workup. This study has many limitations. IgG class resulting in hemolysis, eosin-5′-maleimide (EMA) flow study, genetic analysis to determine hereditary hyperbilirubinemias, and follow-up for the long-term sequel could not be performed. Secondly, the use of the Lui freeze technique of elution could have helped in assessing DAT-negative ABO HDN better.

## Conclusions

A paradigm shift in HDN has occurred over the last few decades following the introduction of standard prophylactic Rh D immunoglobulin administration in Rh D-negative women. ABO incompatibility and other non-D alloantibodies have emerged as the significant causes of immune-mediated HDN in different parts of the world. However, DAT alone is not a reliable marker for detecting HDN, particularly in newborns with ABO incompatibility. Hence, the combined use of DAT with clinical features, peripheral blood smear pictures, hemolytic characteristics, and advanced immunohematological workup like neonatal RBC elution would be more beneficial for predicting the development and severity of HDN. Rh HDN can be detected in Rh D-positive mothers. Therefore, overall screening for both the mother and neonate is recommended for providing optimal treatment, as prevention is always better than cure. However, non-immune causes of HDN are also being ascertained with better molecular understanding and closer interdepartmental liaisons. These conditions have bearings for the future due to their inheritance pattern, and hence an accurate and timely diagnosis is essential in all cases of HDN. A thorough investigation and appropriate follow-up can prevent future neonatal morbidity and mortality.
